# Pulling the Brakes on Fast and Furious Multiple Drug-Resistant (MDR) Bacteria

**DOI:** 10.3390/ijms22020859

**Published:** 2021-01-16

**Authors:** Abid Ali Khan, Khanzadi Nazneen Manzoor, Aamir Sultan, Maria Saeed, Mahrukh Rafique, Sameen Noushad, Ayesha Talib, Simone Rentschler, Hans-Peter Deigner

**Affiliations:** 1 Center for Precision Medicine, Hochschule Furtwangen University, Jakob-Kienzle-Str. 17, 78054 Villingen-Schwenningen, Germany; s.rentschler@hs-furtwangen.de; 2 Department of Biosciences, COMSATS University Islamabad, Park Road, Tarlai Kalan, Islamabad 45550, Pakistan; knazneen02@gmail.com (K.N.M.); rana.amir053@gmail.com (A.S.); maria.saeed93@yahoo.com (M.S.); mahrukhrafique93@gmail.com (M.R.); sameennoushad440@hotmail.com (S.N.); 3 Mechano(bio)chem Department, Max Planck Institute for Colloids and Interfaces, Am Mühlenberg 1, Golm, 14476 Potsdam, Germany; Ayesha.Talib@mpikg.mpg.de; 4 Max Planck Institute of Colloids and Interfaces, Leipzig, Schillingallee 68, 18057 Rostock, Germany; 5 Faculty of Science, University of Tuebingen, Auf der Morgenstelle 8, 72076 Tuebingen, Germany

**Keywords:** multidrug resistance, nanoantibiotics, nanoparticles, combination therapy, bacteriophages

## Abstract

Life-threatening bacterial infections have been managed by antibiotics for years and have significantly improved the wellbeing and lifetime of humans. However, bacteria have always been one step ahead by inactivating the antimicrobial agent chemically or by producing certain enzymes. The alarming universal occurrence of multidrug-resistant (MDR) bacteria has compelled researchers to find alternative treatments for MDR infections. This is a menace where conventional chemotherapies are no longer promising, but several novel approaches could help. Our current review article discusses the novel approaches that can combat MDR bacteria: starting off with potential nanoparticles (NPs) that efficiently interact with microorganisms causing fatal changes in the morphology and structure of these cells; nanophotothermal therapy using inorganic NPs like AuNPs to destroy pathogenic bacterial cells; bacteriophage therapy against which bacteria develop less resistance; combination drugs that act on dissimilar targets in distinctive pathways; probiotics therapy by the secretion of antibacterial chemicals; blockage of quorum sensing signals stopping bacterial colonization, and vaccination against resistant bacterial strains along with virulence factors. All these techniques show us a promising future in the fight against MDR bacteria, which remains the greatest challenge in public health care.

## 1. Introduction

**Figure 1:**
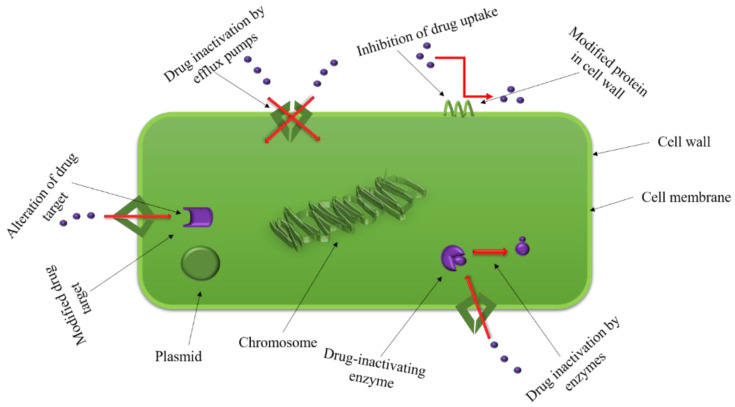
The possible ways a bacterium resists the action of an antibiotic drug.

Infectious diseases around the globe, once cured with the help of the magical drugs “antibiotics”, are now becoming a menace due to ever-increasing microbial antibiotic resistance. This emerging resistance is due to the irrational use of antibiotics in humans, veterinary, and agriculture because of their easy and unregulated access, especially in developing countries. This antibiotic resistance is either acquired naturally or artificially (by transfer of resistance genes) [1,2,3]. The occurrence of antibiotic resistance first became evident when *Staphylococci* encountered the first commercially produced antibiotic, penicillin, which produced an enzyme (penicillinase) to degrade it. The continuous use of diverse drugs has imposed a selective pressure on bacteria, transforming them into “superbugs”, also known as multiple drug resistant (MDR) microorganisms [3]. MDR bacteria that are very difficult to treat include *Pseudomonas aeruginosa*, *Acinetobacter baumannii*, *Escherichia coli*, *Klebsiella pneumoniae*, vancomycin-resistant *Enterococci* (VRE), methicillin-resistant *Staphylococcus aureus* (MRSA), vancomycin-resistant *S. aureus* (VRSA), and extensively drug-resistant (XDR) *Mycobacterium tuberculosis* [4]. Bacteria can exhibit resistance either naturally, i.e., entirely lacking the target or having low-affinity targets; by having the potential to inactivate the antibiotics; low cell permeability and the presence of efficient efflux pumps, by the transfer of resistant genes found on plasmids, transposons, and bacteriophages [1,2]. Different sophisticated mechanisms have been evolved by bacteria for resistance to antibiotics and to protect themselves from being killed by these antimicrobial agents [5]. Among these different mechanisms, one of the most successful processes is to inactivate the antimicrobial agent by chemically altering or destroying it. It is accomplished by producing certain enzymes, e.g., aminoglycoside modifying enzymes that modify the amino groups of aminoglycoside molecules, β-lactamases that destroy the amide bond of the β-lactam ring of β-lactam antibiotics [6]. Another mechanism of resistance is by decreasing the influx of antimicrobial agents in Gram-negative bacteria due to the presence of outer membrane, i.e., vancomycin resistance [7,8,9]. Efflux pumps present in bacteria are capable of secreting the antibiotic out of the cell are also one of the reasons for resistance to antimicrobial agents [10]. Another common strategy of resistance is to prevent antibiotic action by interfering with the target site in several ways like protecting (tetracycline and fluoroquinolones resistance) or modifying the target site (rifamycin resistance). Much astonishing diversity is present within these categories of mechanisms, and several types of resistance may be possessed by a single strain [5,11]. The general modes of resistance in bacteria are diagrammatically shown in Figure 1. There are several mechanisms to prevent and combat these MDR bacteria. One of these includes nanoparticles (NPs) acting as a weapon against emerging antibiotic resistance. NPs (and nanoencapsulation platforms), due to their high surface area to volume ratio and functionalizable structural surface(s), can effectively interact with microorganisms causing fatal changes in the morphology and structure of these cells [12,13,14]. Nanophotothermal therapy is an approach in which inorganic NPs such as AuNPs can absorb magnetic radiation and convert it into heat, which can destroy pathogenic bacterial cells in the close locality. MDR bacteria are effectively killed by this technique [1,15,16,17,18]. Bacteriophage therapy is another mechanism to prevent the appearance of these lethal microorganisms. It is no less than a magical cure for many antimicrobial-resistant infections [3]. Bacteriophages are self-replicative, and bacteria develop less resistance against them when compared with the usage of antibiotics. To treat MDR bacteria, a suitable cocktail of phages is required [19].

**Figure 2:**
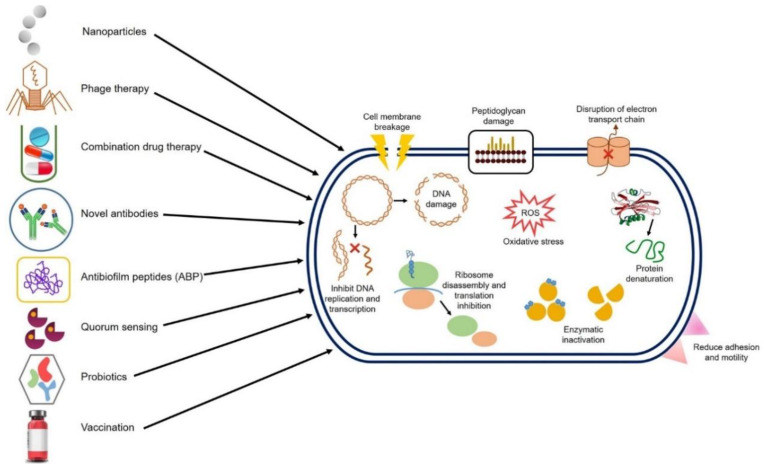
A pictorial presentation of various strategies, targets and effector molecules that can be used to curb the multiple drug resistance in bacterial pathogens.

Combination drugs that act on different targets in different pathways can be used in such a way that even if a bacterium is resistant to one of the drugs, the other drugs targeting different components of bacteria will disrupt them, thus minimizing their propagation [3]. Probiotics can also prevent antibiotic resistance by the secretion of antibacterial chemicals, diminishing the ability of bacteria to colonize the body, thereby reducing the use of antibiotics and hence the emergence of MDR [20,21]. Bacteria communicate with each other through quorum sensing; blockage of these signals would prevent bacterial colonization and therefore considerably reduce the need for antibiotics [22]. Vaccines lower the disease incidence and, concurrently, the need to use antimicrobial drugs. There is a potential prospect to develop vaccines against resistant bacterial strains as well as against virulence factors to step up the game against MDR bacteria [23]. Vaccines may be regarded as being superior to drugs due to being prophylactic and expressing multiple epitopes [24]. Figure 2 represents the different strategies and targets we can employ to fight multidrug resistance in bacteria.

## 2. Nanoparticles as a Weapon against Antibiotically Resistant Bacteria Extracorporally

Bacteria are responsible for causing a great variety of ailments each year around the globe, and the rapidly growing antibiotic resistance has caused an alarming situation in the field of medical microbiology. Infections due to MDR bacteria pose a great threat because they cause chronic disease states resulting in high rates of mortality, morbidity, and prolonged treatment costs [25]. MDR bacteria that are most problematic to deal with are XDR *M. tuberculosis*, *A. baumannii*, *P. aeruginosa*, MRSA, *E. coli*, and *K. pneumoniae* bearing NDM-1 (New Delhi metallo beta-lactamase-1), VRE, and VRSA [1,26,27]. NPs may serve as an imperative tool for fighting antibiotic resistance [28]. The general mode of antimicrobial action of different NPs is shown below, which is based on their exceptionally large surface area and functionalizable structure that enables them to effectively interact with microorganisms causing changes in the morphology and structure of bacterial cells (Figure 2) [1]. The basic mechanism includes anchoring the cell wall of a bacterium and penetrating it, altering the cell membrane permeability, or making it porous by the production of free radicals, ultimately causing cell death [1,16,27,29].

Metallic NPs cause bacterial cell death by interacting with the sulfur and phosphorus in the bacterial DNA bases, thereby destroying the DNA. NPs can also inhibit signal transduction and, consequently, bacterial growth by the dephosphorylation of peptide substrates on tyrosine residues [1,30,31]. Certain NPs can destroy the membrane potential and suppressing the ATPase activities to reduce the levels of ATP in the cell, while others by inhibiting ribosomal subunit from binding the tRNA molecules [1,32,33]. Li et al. reported that 2 nm AuNPs with cationic surface chemistry could interact with the cell membrane of Gram-positive and Gram-negative bacteria resulting in the formation of distinct aggregation patterns and promote bacterial cell lysis [34,35]. Correspondingly, Jiang et al. also established that bacterial cell membrane could be damaged by cationic AuNPs induced protuberance [35,36].

### 2.1. Metal Oxide, Nitric Oxide, and Chitosan NPs

**Figure 3:**
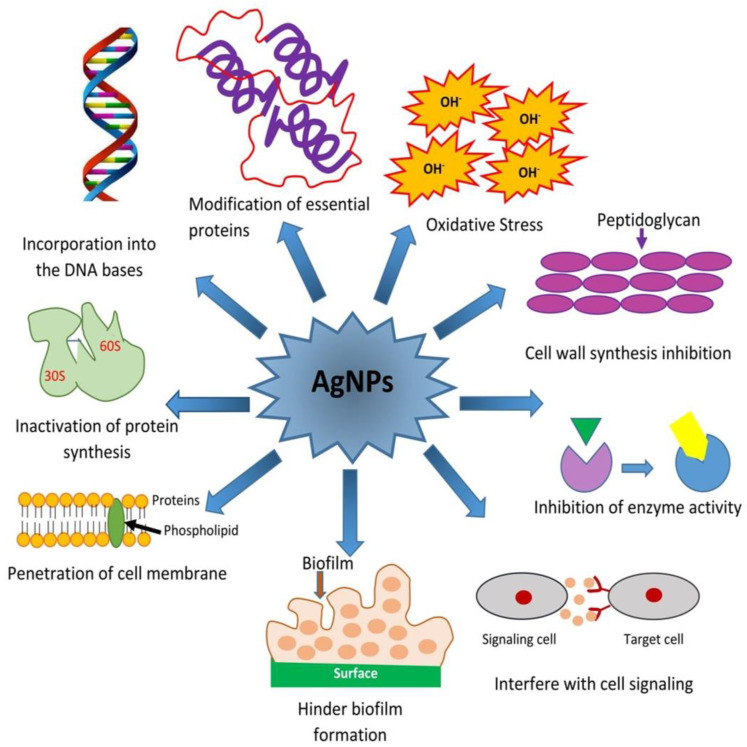
Different modes of action possible for the eradication (killing) of bacterial cells through silver (Ag) nanoparticles [1].

Metal oxide NPs such as TiO_2_, CuO, and ZnO, by the production of reactive oxygen species (ROS), also act as antibacterial agents against MRSA and *E. coli*, but their efficiency is increased when they are coupled with AgNPs. They have photocatalytic activity due to a wide bandgap, which is attributed to the production of ROS [1,37,38]. CuONPs have shown to be effective against a variety of bacterial pathogens, including MRSA, *E. coli*, *S. aureus*, *P. aeruginosa*, *N. meningitis*, *B. cereus*, *S. pyogenes*, and *A. baumannii* [1,26,39,40]. The mode of action of AgNps is shown in Figure 3. When CuONPs are conjugated with AgNPs, their antibacterial activity is enhanced so that they can completely inhibit bacterial growth. ZnONPs have shown acute toxicity to antibiotic (methicillin)-resistant bacteria such as *S. aureus* and *S. agalactiae*. ZnONPs are internalized into the cells where they disorganize and damage the cell, cell membrane and also increase the oxidative stress that damages bacterial proteins, lipids, and DNA [1,41,42]. However, at low concentration, ZnONPs show slight toxicity indicating that the level of toxicity caused by these NPs depends upon their concentration. The colloidal suspension of ZnO is found to inhibit 90% of MRSA, *E. faecalis*, a high biofilm-producing strain *S. epidermidis*, and the growth of several other clinically relevant pathogens. ZnONPs were also shown to inhibit bacterial growth of methicillin-sensitive *Staphylococcus aureus* (MSSA), MRSA, and methicillin-resistant *Staphylococcus epidermidis* (MRSE) strains. Moreover, these NPs were also found to be effective against extended-spectrum β-lactamases-producing *E. coli* and *K. pneumoniae* apart from other bacteria like *Vibrio cholera* and *Campylobacter jejuni* [1,43,44,45,46].

Nitric-oxide-releasing NPs (NONPs) also act as antimicrobial agents against many antibiotic-resistant and sensitive bacteria, i.e., *K. pneumoniae*, *E. faecalis*, *S. pyogenes*, *E. coli*, and *P. aeruginosa*. NO is unstable in the presence of oxygen and reacts with oxygen or superoxide spontaneously to produce reactive nitrogen and oxygen intermediates that are toxic against cells and act as antimicrobial species. When the concentration of NO is greater than 1 µM, these intermediate species become significantly important because, at these concentrations, reactive nitrogen species (RNOS) like S-nitrosothiols (RSNO), peroxynitrite (OONO−), nitrogen dioxide (NO_2_) are produced [1,47,48]. Peroxynitrite and nitrogen dioxide have demonstrated NO-associated lipid damage, which shows the antimicrobial activities associated with NO [1,49,50].

NO interactions with proteins involve reactive thiols, heme groups, iron-sulfur clusters, phenolic or aromatic amino acid residues, tyrosyl radicals, or amines. Peroxynitrite and NO_2_ also nonspecifically oxidize proteins at many sites. NO can also inactivate the enzymes containing Fe-S clusters (e.g., aconitase, NADH dehydrogenase, succinate dehydrogenase), thereby suggesting that NO• (NO radicals) may cause the release of iron from metalloenzymes and result in iron depletion [1,51,52]. The enzymes DNA alkyl transferases have cysteine residues where the -SH group of cysteine residues reacts with NO, resulting in the formation of S-NO adducts. These adducts, in turn, inhibit the transfer of the alkyl group from guanine to the protein. Thus, NO inhibits DNA repair enzymes, which are particularly concerned with the repair of alkylation to DNA [53,54]. The sensitivity of prokaryotes to NPs treatment is higher because bacteria depend a great deal on iron-sulfur clusters as compared to mammalian cells. Consequently, it seems reasonable that efficient NO-releasing NPs have the potential to be effective against MDR bacteria [1,55]. The in vitro efficacy of NONPs has been assessed against several clinically significant Gram-positive (*E. faecalis* and *S. pyogenes*) and negative (*E. coli*, *K. pneumoniae*, and *P. aeruginosa*) isolates. It was found that the reduction in bacterial growth was NONPs dose-dependent for both Gram-negative as well as Gram-positive bacteria. The growth of Gram-negative bacterial isolates was inhibited within 24 h by NONPs; however, Gram-positive bacterial growth was inhibited within 8–16 h with lower NONPs concentrations [1,56,57].

Chitosan NPs are known to be suitable for non-invasive routes of drug administration (nasal, oral, ocular, and pulmonary routes) as they deliver the drug with reduced toxicity, increase the blood half-life of drugs as well as the efficiency of intravenous injections [58,59]. Biofilms produced by *P. aeruginosa* are one of the main challenges while treating (the skin) infections. In one study, gold nanorods (AuNR) were decorated with 1,2-Distearoyl-sn-glycero-3-phosphorylethanolamine(DSPE) phospholipids and exploited to destroy biofilms produced by *P. aeruginosa* In vitro. Roughly a ~6 log cycle reduction of the bacterial count was observed by applying DSPE-AuNR against *P. aeruginosa*, proving the fact that gold-based nanosystem is one of the effective alternatives to antibiotics for the eradication of biofilms [60]. In another report, the effect of hyperthermia of gold nanorods (GNR) against *S. aureus* and *Propionibacterium acnes*, the causative agents of acne vulgaris, were evaluated. Local heat was generated when functionalized GNR was excited by a laser beam. It resulted in a ≥99.99% reduction of viable bacterial count [61].

### 2.2. Nano-Photothermal Therapy of MDR Bacteria

Another technique employed for destroying biological cells is Photothermal therapy with NPs. In this technique, the electromagnetic radiation absorbed by the NPs is converted into heat, which is then transferred via thermal conduction to the bacteria or cells in close proximities [1,18,62]. AuNPs have been extensively studied for photothermal therapy of cancer [1,63,64,65]. It has been shown that pathogenic bacteria can also be selectively killed by using functionalized AuNPs. AuNPs, when conjugated with vancomycin, acquire a polygonal shape, due to which these NPs have the potential of absorbing near-infrared (NIR) light. Amoxicillin coated Au-NPs have increased in vivo stability [1,66,67]. NPs conjugated with vancomycin can effectively kill bacterial cells under illumination (>99%). It has been established that pathogenic *E. coli* can be photothermally lysed using Au nanorods [1,29,68], and *P. aeruginosa* can also be effectively killed by the same metallic nanorods conjugated with primary antibodies [1,69].

The bacterial cell viability also reduces significantly when a nanorod attaches to the surface of the bacterial cells and is exposed to near-infrared radiation. In addition, the MDR bacteria are photothermally destroyed by multifunctional popcorn-shaped magnetic iron core-shell gold nanoparticles. Results had shown that when MDR *Salmonella* DT104 bacterial cells were treated with M3038 antibody-conjugated hybrid platforms, they attached to bacterial cells, and localized heating at 670 nm light irradiation caused irreparable cellular damage and killed the bacteria within 10 min of exposure [70]. MRSA and *E. coli* had been reported to show a significant decrease in viable counts when exposed to 660 nm for 5 minutes, along with polysiloxane polymers containing embedded methylene blue and AuNPs [1,71,72].

### 2.3. Silver NPs Bactericidal Effect against Multidrug-Resistant Bacteria

**Figure 4:**
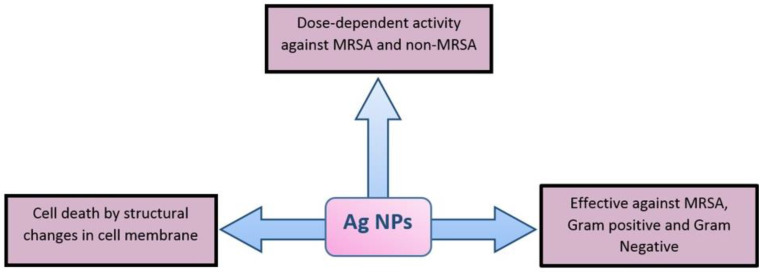
Mode of action (bactericidal effects) of AgNPs against bacteria against MRSA.

Historically silver has been used for its antiseptic and bactericidal activity in dental alloys and open wounds as well as in Ayurveda and homeopathy. Gram-positive bacteria possess a thick peptidoglycan layer (30 nm) in comparison to Gram-negative bacteria (2–3 nm), and silver NPs are thought to anchor the cell wall leading to structural changes in the cell membrane, thus increasing the cell permeability. Therefore, uncontrolled transport through the cell membrane results in bacterial cell death [34]. In addition, AgNPs produce free radicals for membrane damage and may affect the proton motive force inhibiting the oxidative phosphorylation [55]. AgNPs can release Ag^+^ ions, which can disrupt cellular functions by interacting with thiol groups of many enzymes rendering them inactive [1,73,74]. Panacek et al. established that the colloidal AgNPs have substantial bactericidal activity against MRSA, Gram-positive and Gram-negative bacteria [55]. Ayala-Nunez et al. observed that the mode of action of AgNPs (100 nm size) is dose-dependent against MRSA and non-MRSA as their growth was inhibited at concentrations over 1.35 mg/mL when the inoculum was 10^5^-CFU/mL [55,75]. Nanda and Saravanan reported the antimicrobial activity of AgNPs synthesized by aqueous Ag^+^ reduction with *S. aureus* against MRSA, MRSE, *S. pyogenes*, *Salmonella typhi*, and *K. Pneumoniae* and reported that AgNPs were most effective against MRSA followed by MRSE and *S. pyogenes*, but only moderate activity was observed against *S. typhi* and *K. pneumoniae* [55,76]. Humberto et al. found that AgNPs of concentration 30 to 100 mmol/L is effective against the erythromycin-resistant *S. pyogenes*, ampicillin-resistant *E. coli*, MDR *P. aeruginosa*, and drug-susceptible strains including *Streptococcus* spp., *E. coli*, and *P. aeruginosa* [55,77]. Pal et al. found that triangular AgNPs are more active than spherical NPs, which are again more active than rod-shaped NPs against *E. coli*. Morones et al. studied the effect of different concentrations of AgNPs (1–100 nm size) on *E. coli* and concluded that concentration over 75 µg/mL was sufficient for a significant decline in the bacterial progression. Shrivastava et al. found that the mode of action of AgNPs is dose-dependent and is more evidence against Gram-negative bacteria as compared to Gram-positive bacteria [55]. Recently, our group reported that AgNPs synthesized through green routes can check many susceptible and MDR bacteria very effectively [26]. The bactericidal effect of AgNPs against MDR bacteria is illustrated in Figure 4.

The antibacterial activity of NPs is directed by their physicochemical properties, which in turn depend upon the route(s) of synthesis [1,78]. It has been reported that NPs could be synthesized by Fungi like *Fusarium oxysporum* and *Phanerochaete chrysosporium*. Magnetotactic bacteria are also well known to biosynthesize magnetic iron oxide nanoparticles [79,80]. Biosynthetically produced AgNPs using fungus, yeast, bacteria, and plant extracts were found to have strong antibacterial efficacy against various MDR pathogens such as *M. tuberculosis*, *P. aeruginosa*, *S. pneumoniae*, MRSA, *K. pneumoniae*, MRSE, *S. pyogenes*, *Bacillus* spp., *E. coli* and *S. typhi* [1,77,81,82]. Green NPs have improved antibacterial activity because of their high surface area to volume ratio and surface reactivity as compared to the chemical NPs. In addition, the coating of biosynthetically produced NPs by phytochemicals increased their ability to inhibit bacterial growth in comparison to chemical NPs [1,83,84].

### 2.4. Aluminum Oxide Nanoparticles

The antimicrobial effects of metallic NPs, particularly AgNPs, have been extensively reviewed, but there is not enough substantial literature regarding the antimicrobial activities of Aluminum oxide NPs. Aluminum oxide NPs, also called alumina, are normally known as corundum, which is the crystalline form of alumina (Al_2_O_3_) [33]. They adopt a corundum-like structure as oxygen forms a hexagonal packing, and Al^+3^ fills two-third of the lattice in the octahedral sites [85]. Alumina NPs possess a positive charge on their surface at almost neutral pH and are thermodynamically stable over a wide range of temperatures. They can disrupt the bacterial cell wall by producing ROS resulting in bacterial cell death [86]. Among the various diverse procedures for the synthesis of Alumina NPs, for instance, hydrothermal processing, sol–gel pyrolysis, sputtering; the laser ablation method is widely used as it is a quick and high-purity process [87]. Aluminum NPs have been documented to have a wide range of antimicrobial activities. Their growth-inhibitory effects on *E. coli* have been reported at 10–1000 µg/mL [88]. Balasubramanyam et al. have reported high sensitivity of Alumina against *P. fluorescence* as compared to the bulk materials [89]. In one study, aluminum oxide NPs were proven to be a good antibacterial agent against Gram-positive as well as Gram-negative bacteria. These NPs were synthesized by using aluminum sulfate and NaOH as precursors by the co-precipitation method. Their activity was analyzed against Gram-positive (*S. aureus* and *Streptococcus mutans*) and Gram-negative (*E. coli* and *Proteus vulgaris*) bacteria [90].

An oxide layer is produced over alumina nanoparticles, which then protects these from oxidation. These particles are affected by different pH concentrations, which lead to their different toxicity levels; for example, at neutral pH, these NPs have a positive surface charge due to which they would have an affinity towards the negatively charged surface of *E. coli* cells, thereby resulting in adhesion of alumina NPs over the bacterial surface. Moreover, these NPs also can serve as radical scavengers and cause distortion in bacterial cells [91].

### 2.5. Silicon NPs

Silicon dioxide (SiO_2_) is one of the important industrial additives with several applications. It is commonly used as a semiconductor in electronics when in the crystallized form [92]. The conversion to metal oxide increases the surface area, thereby providing better performance in applications. Jiang et al. have shown the binding of SiO_2_NPs to the bacterial cell walls as well as their higher toxicity as compared to their bulkier counterparts [93]. As limited data are available regarding the antibacterial activity of SiO_2_NPs, more research is needed to be done to understand their clear role. For treating multidrug-resistant *M. tuberculosis*, ethionamide (ETH) is one of the most important drugs used. In one study, it was reported that loading ETH into thermally carbonized-porous silicon (TCPSi) nanoparticles would result in enhancement of solubility and permeability of ETH at different pH-values as well as increased its metabolization process. It was found that ETH-conjugated SiNPs tend to reduce the dosing frequency of ETH for the treatment of multidrug-resistant *M. tuberculosis* [94]. Moreover, it was demonstrated in a study that porous silicon nanoparticles have the potential to be used as a means of a prolonged drug delivery system [95].

### 2.6. Gallium Nanoparticles (NPs)

There is a growing problem of drug-resistant strains of *M. tuberculosis*, due to which there is an urgent need for new treatments and novel drugs. In one study, targeted drug delivery using gallium (III) nano-formulations were used against drug-resistant *M. tuberculosis*, which showed promising anti-tuberculous activity. They also promoted maturation of the phagosome, which in turn result in the increased macrophage-mediated killing of the organism.

*M. tuberculosis* requires iron for growth and replication, gallium encapsulated in nanoparticles interferes with the cellular iron acquisition and utilization, which in turn inhibit the growth of this bacteria human monocyte-derived macrophages (MDMs). Delivery of Ga in the form of nanoparticles to macrophages open new pathways for the development of new therapeutic anti-tuberculous drugs [96]. Similar to *M. tuberculosis*, iron and gallium encapsulated in nanoparticles can also be used against HIV. GaNPs are readily internalized by the MDMs, and then sustained drug release causes significant growth inhibition of both HIV and *M. tuberculosis*. Iron-mediated enzymatic reactions are interrupted by GaNPs, which leads to growth inhibition of HIV–*M. tuberculosis* coinfection in macrophages, and it also modulates the release of cytokines that may contribute to HIV-TB pathogenesis [97]. Table 1 summarizes different nanoparticles that can be exploited as a weapon against multidrug-resistant bacteria.

## 3. Host Defense Peptides (HDPs)

The MDR infections are burgeoning at an alarming rate, and hardly any discoveries are taking place in the manufacturing of novel antibiotics to treat such stubborn bacterial infections. The seriousness of this matter creates a need for substitute strategies to treat bacterial infections. Host defense peptides (HDPs) are considered as some effective alternates that would help fight the resistant bacterial infections. These short cationic molecules are formed by the immune systems of many multicellular organisms. The evolution in nature has resulted in the formation of some remarkable HDPs, which possess diversity in structure as well as in biological activity. These natural peptides can be used as templates to generate a single synthetic molecule having the combined properties of antimicrobial and immunomodulatory compounds to fight the resistant bacteria when existing antibiotics fail to function. Defensins and cathelicidins are the two main families of the naturally existing HDPs [100].

Two novel peptides, brevinin1 HYba1 and brevinin1 HYba2, had been isolated from frog (*Hydrophylax bahuvistara*) skin secretions, and their hemolytic, cytotoxic, and antibacterial activities were investigated after designing acidic and amidated analogs. All the peptides, excluding acidic analogs, showed promising antimicrobial activity against tested Gram-positive and Gram-negative bacteria. These peptides also showed very low hemolysis on human erythrocytes. This study opened up an area to explore more natural sources of host defense peptides, which can be used as effective therapeutic agents [101].

## 4. Defensins

**Figure 5:**
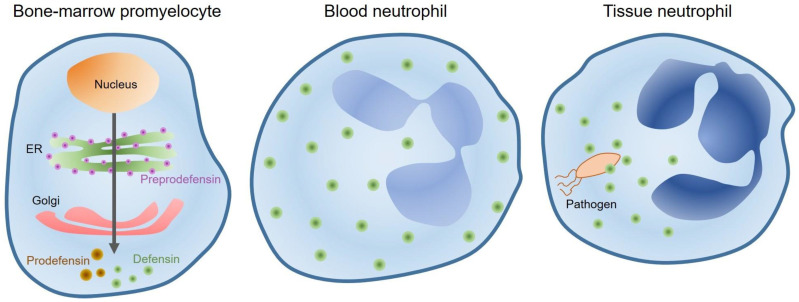
Promyelocytes in the bone marrow synthesize α-defensins. 94 amino acid preprodefensin (purple) is biosynthesized in the ribosomes; the 19 amino-acid N-terminal signal sequence is cleaved and it converted to a 75 amino-acid prodefensin (brown). Subsequent cleavage of residues generates a 29–30 amino acid mature defensin (green). During phagocytosis (pathogens), defensin-rich primary granules fuse with phagocytic vacuoles and high concentrations of defensins are generated.

Defensins are cationic amphipathic peptides having approximately 30 amino acid residues. The three disulfide bonds in the structure stabilize the triple-stranded antiparallel β-sheet assembly [102]. Based on the arrangement of disulfide bonding, defensins are further divided into subfamilies: α, β, and θ. Among mammals, only human neutrophils and leukocyte granules possess α-defensins [103]. In most mammals, Paneth cells of the intestines are responsible for the production of these defensins [104]. They are synthesized as precursors initially and can become activated when the N-terminal segment is removed with the help of trypsin in humans (Figure 5). The concentration of α-defensins reaches 10 mg/mL when they are stimulated by microbes. This concentration is enough to tackle a resilient microbial infection [100].

Most of the epithelial cells express β-defensins; proinflammatory stimuli and infections are responsible for this expression of β-defensins. They can be found in mucosal sections of gastrointestinal, respiratory, and urogenital tracts as well as in the inflamed skin [100].

The θ-defensins are the rarest of all the three defensins and are cyclic molecules. Due to the cyclic structure of θ-defensins, their microbicidal action is resistant to the concentration of salt. θ-defensins are absent in mammals, including humans.

A variety of different defensins have also been identified in different fungi. In one study, 68 fungal defensin-like peptides (fDLPs) from five genera named *Apophysomyces*, *Trichosporon*, *Scedosporium*, *Beauveria*, and *Lichtheimia* had been reported. A new synthetic defensin called scedosporisin had been characterized, which shows good activity against Gram-positive bacteria. It killed several vancomycin-resistant *Enterococci* and MRSA while it showed less cytotoxicity and hemolysis. It was found out that scedosporisin-2 killed bacteria more rapidly as compared to the antibiotic vancomycin [105].

## 5. Cathelicidins

Cathelicidins are the second major group of HDPs and are categorized based on a production mechanism rather than a sequence match. The inactive precursors of cathelicidins consist of N-terminal cathelin-like domain, which is followed by a peptide region. These precursors are proteolytically cleaved to become mature and active HDPs [106]. Cathelicidins differ in sequence, length, as well as in structure. They have lengthy α-helical and β-hairpin folds along with some short linear molecules. These short 13 amino acid molecules are the initiators for designing synthetic peptides that have optimized biological activity.

Many types of cathelicidins, such as bactenecin, indolicidin, protegrins, and many others, are produced by the immune systems of bovine and porcine [106]. The human immune system is known to produce only one type of cathelicidin precursor protein hCAP18. This precursor is processed proteolytically to produce a mature cathelicidin LL-37 [107]. Disulfide bonds are absent in LL-37. However, it adopts the conformation of α-helical when it interacts with the lipid bilayers. Mice are known to have only one cathelicidin precursor, which is also proteolytically cleaved to produce a mature form, CRAMP. This mature peptide has a sequence identity of 67% with LL-37 [100].

*S. aureus* is responsible for many serious infections in humans that sometimes lead to sepsis or death also. In one study, six novel cathelicidins named CATHPb1–6 were identified from *Python bivittatu*. CATHPb1 was found with an excellent pharmacological and toxicological profile In vitro. Later on, it had been observed that CATHPb1 provides efficient protection to mice against MRSA/VRSA. CATHPb1 was found to be involved in rapidly modulating macrophages/monocytes as well as trafficking the neutrophils to the site of infection and also enhance their bactericidal functions. It also increases the levels of chemokines and reduces the release of proinflammatory cytokines. Therefore, it proved to be a novel therapeutic agent against MDR *S. aureus* [108]. Table 2 shows HDP and their target bacteria.

## 6. Antimicrobial Peptides (AMP)

Antimicrobial peptides (AMP) are evolutionarily conserved macromolecules produced by most living organisms ranging from prokaryotes to humans as a first line of defense. AMP are a part of innate immune response and have an ability to fight against pathogenic microbes. These small peptides ranging from 05 to 100 amino acid residues are generally cationic in nature and are folded into unique structures that facilitate their mode of action. AMP help to eradicate (pathogenic) bacteria either by killing them directly or by modulating the host immune response. They can be used against a number of microbes and have proven to be a promising agent when used as antibacterial either alone or in combination with other methods [102,106,109].

Antimicrobial photodynamic therapy is one of the novel approaches, which, in combination with antimicrobial peptides, has a great potential to act as a favorable tool against MDR bacteria. Photodynamic therapy is quite effective against Gram-positive bacteria, but combinational therapy with AMP makes it potent against Gram-negative too. Photodynamic AMP generate reactive oxygen species upon exposure to light of a certain wavelength and disrupt the cell wall and membrane resulting in cell death [110].

**Figure 6:**
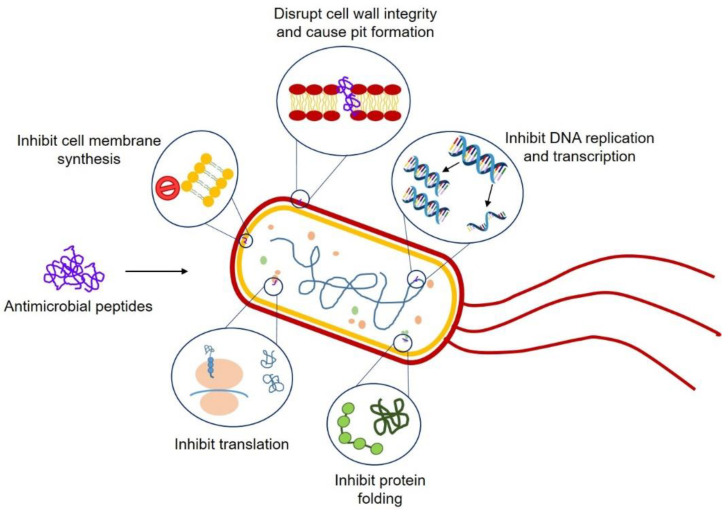
Possible mechanisms antimicrobial peptides can kill bacterial superbugs.

Biofilms are three-dimensional multicellular structures that form on natural and/or clinical surfaces. Biofilms are adaptively resistant to antibiotics, due to which they are difficult to treat as compared to their planktonic forms [111]. They are formed on various implanted devices, and these aggregates can only be removed by surgery [112]. Therefore, it is very challenging to treat biofilm-related infections; there is an urgent need for new therapeutic options to fight them out. In recent years, various approaches had been developed, such as bacteriophages, antibodies, quorum sensing antagonism, etc. One of the most promising approaches is the use of antibiofilm peptides (ABP), which are a class of the AMP; these proteinaceous entities can either be cationic or amphipathic [113] and are a part of the host defense peptides. The first-ever cationic peptide, nisin, was isolated in 1928 from *Lactobacillus lactis*. It was relatively stable at room temperature, but at pH 2–6, it showed high antimicrobial activity [114,115]. Natural AMP polylysine was isolated from *Streptomyces albulus* 346 and is now commercially produced for a variety of food applications as preservative agents [116]. ABP shows activity against a variety of resistant Gram-positive and Gram-negative bacterial strains. They are also found to be very effective against fungal microbes. Interactions of these peptides with bacterial components do not require any specific protein binding sites, and it is why they would theoretically not have any resistance emerged against them [109]. Recently, they have been put to work in many applications, including oral candidiasis, catheter-associated, and implant surface infections [117,118]. The human cathelicidin peptide LL-37 was shown to exhibit antibiofilm activity against *S. aureus* and *E. coli* [119]. The two tryptophan-rich cationic AMP KT2 and RT2 were found to show antibiofilm activity against enterohemorrhagic *E. coli* O157:H7, which is a multidrug-resistant strain. These two peptides did not only prevent the biofilm formation but also could eliminate mature biofilms [120]. It has also been reported that these peptides can be used in conjugation with other antimicrobial compounds to enhance their activity (synergistic effects) [121]. This synergy helps in lowering the concentration of antimicrobial compounds, which will, in turn, reduce the (toxic) side effects of these compounds and stop the spread of antimicrobial resistance [122]. Most commonly, these peptides permeabilize bacterial cell membranes, which lead to the death of cells either by causing large damage or small obstructions that will, in turn, disturb transmembrane potential leading to cell death [123]. Specifically, their mechanism of action has been explained by pore and non-pore models. For pore models, there are two theories; the toroidal pore model, in which antimicrobial peptides can affect the curvature of membrane and the barrel stave pore model, in which these will interact with the cell membrane and form a hydrophilic pore [124,125]. For non-pore models, there are many theories, such as the detergent model, the molecular shape model, and the carpet model [126]. Among these, the carpet model is the most common model. According to this model, peptide monomers form a layer on the surface of the membrane, which leads to the destabilization of a phospholipid bilayer, which then results in the breakdown of the membrane [127]. Much research has been carried out to develop antimicrobial peptides as effective antimicrobials, but hurdles are there because these have complex interactions with membranes as well as with each other. Very limited data are available on such peptides having antibiofilm properties. Therefore, more work is needed to be done to understand the proper mechanism of action [128]. However, we have shown various possible ways ABPs can get over a superbug and kill it when it resists antibiotic drugs in Figure 6.

ABP can also be obtained from the poisons of various animals like ants, wasps, bees, scorpions, and spiders. One such peptide, called Mastoparan peptide, isolated from Vespidae venom showed broad-spectrum antimicrobial activity against both Gram-positive and Gram-negative bacteria, *Mycobacteria* and fungi. In one study, two peptides, agelaia-MPI and polybia-MPII, isolated from wasps, showed bactericidal activity along with antibiofilm activity against biofilm-forming MDR *Acinetobacter baumannii* [129]. Five ocellatin peptides, ocellatin-PT2–PT6, had been isolated from frog *Leptodactylus pustulatus* skin secretion and used against an MDR opportunistic pathogen, *P. aeruginosa*, where they effectively killed the bacterial pathogen. Another ocellatin peptide, named ocellatin-PT3, inhibits the proliferation of established biofilms by direct killing of bacterial cells within biofilm [130].

The EPS of some bacterial species like non-typeable *H. influenza*, *S. enterica* serovar Typhimurium/Typhi and *P. aeruginosa* are responsible for the resistance against innate immune components, including AMP. This resistance is particularly due to the structure of the biofilm community. The polysaccharides and extracellular DNA (eDNA) of EPS bind the AMPs because of charge differences [131]. There are specific sensors in bacteria that are responsible for activating the resistance mechanisms against AMP upon exposure [132,133]. It had been demonstrated that in *P. aeruginosa*, the *psrA* gene encodes a transcriptional regulator which upregulates in response to the presence of subinhibitory concentrations of cationic AMP [134]. Table 3 describes possible mode(s) of action of (AMP) ABP with their advantages and limitations.

## 7. Bacteriophage Therapy

Bacteriophages are diverse non-living biological entities that consist of DNA or RNA surrounded by a protein capsid. They are capable of reproducing independently and are ultimately dependent on bacterial hosts for survival. Phages normally bind themselves to specific receptors on the bacterial cell surface, release their genetic material into the host cell and then either incorporate this material into the bacterial genome and reproduce vertically from mother to daughter cells or invade the bacterial replication mechanism to produce the next-generation of phage offspring and lyse the cell. When a critical mass of phage offspring is reached, which can be from a few to over 1000 viral particles, depending on environmental factors, the lytic proteins are activated and hydrolyze the peptidoglycan (cell wall) of bacteria [137]. Scientists suggested that phages can be used as suitable antibiotic agents with having maximum efficiency.

**Figure 7:**
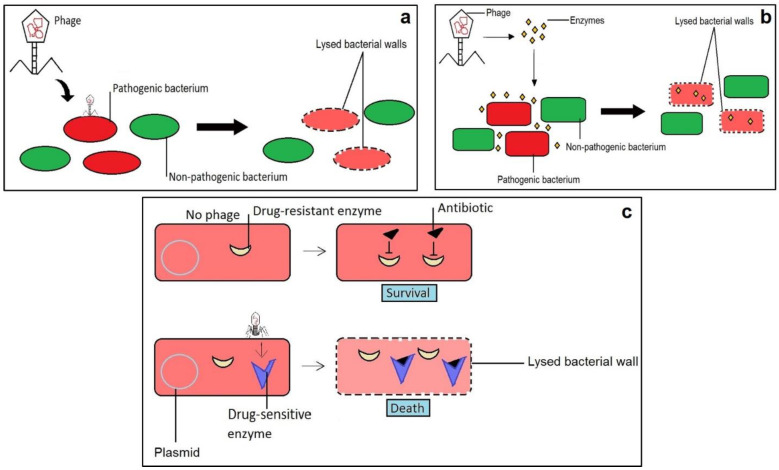
Phage-derived antimicrobial techniques. Novel antimicrobial strategies derived from phages and their products. (**a**) Phages target specific bacterial pathogens and thus cause the lysis of that particular bacterial cell wall; (**b**) phages produce enzymes that target particular bacterial pathogens; (**c**) phages can be used to transfer antibiotic-sensitive genes into drug-resistant bacteria.

Treatment of infectious diseases is becoming difficult and a threat to mankind due to a rise in antibiotic-resistant microbial strains [19]. To prevent and treat such resistant strains, phage therapy is becoming popular and gaining interest all over the world [138,139]. Pioneering (novel) antimicrobial approaches using phage products, or genetically manipulated phages, are being exploited to cope with bacterial infections and antibiotic resistance (Figure 7) [139]. Phages infect bacterial cells and produce endolysins that damage the bacterial cell wall by hydrolyzing the four main bonds of its peptidoglycan constituent in the lytic cycle [138,140,141]. An osmotic imbalance is the cause of lysis when the cell losses structural integrity upon peptidoglycan degradation. In the case of Gram-negative bacteria, the outer membrane is ruptured with the help of complexes (spanins), fusing both the inner and the outer membrane [139].

Bacteriophages have been successfully used against bacterial biofilms, in therapeutics (genetically modified form), in the food industry to minimize the bacterial load and to improve antibiotic potency (Figure 7c) [138,142]. Antibiotics delivered along with the phages permit delivery to specific cells and can cause an upsurge in local drug concentrations [139].

Bacteriophages are used both externally and internally to treat diseases that could otherwise not be cured by antibiotics [138]. For example, in a study, phage application significantly decreased the concentration of bacterial cells (in all patients’ sputum samples), improving overall health. Birds had also been reported to have a reduction in a load of *Salmonella* and *Campylobacter* in the poultry meat when a multivalent/cocktail of lytic bacteriophages was used to help the meat industry to produce safe and good quality edible products [138,143].

Bacteriophages have several advantages over antibiotics, i.e., they have an affinity for a specific bacterium, which helps in typing of that particular bacterium and causes its lysis, whereas using the (broad-spectrum) antibiotics would also harm the normal flora. Due to their replicative nature, there is no need to administer the bacteriophages repeatedly, and Most of the phages can be ingested as they can survive in the gastric environment [140,144] and are even lethal to MDR bacterial superbugs: *E. faecalis*, *S. aureus*, *Klebsiella*, *A. baumannii*, *P. aeruginosa*, and *Escherichia coli* [19]. Because of minimal side effects, they are also considered to be ecologically safe (i.e., harmless to humans, plants, and animals) [19,138,145].

However, the disadvantages of phage therapies must not be overlooked either. In the case of mixed infections, phages are not very effective due to their narrow host range, whereas broad-spectrum antibiotics work effectively in similar scenarios [138,146]. If the selected phage switches to the lysogenic life cycle, it will integrate its genome in the host cell leading to failure in phage therapy. Moreover, if phages are not sequenced before their administration in humans or animals, they may carry a toxic (deleterious) gene that can be harmful [138]. We should always use a cocktail of phages so that the risk of resistance development could be reversed or diminished [19,147].

The frequency of bacterial resistance to phages is significantly lower (10^−7^ to 10^−8^ per cell) compared with that of the resistance to antibiotics (frequency of mutation for one specific gene is 10^−5^ per cell). Bacterial cells can also become resistant to phages as their cell receptors are specific to different phages; constructing the “suitable” cocktail will help to achieve the maximum effectiveness of phage therapy [19].

Recent investigations using animal models have explored phage treatments against different bacteria, which have shown positive results. When challenged with gut-derived sepsis due to *P. aeruginosa*, oral administration of phages saved 66.7% of mice from mortality in comparison to 0% in the control group. In a hamster model of *Clostridium difficile-induced* ileocolitis, a single dose of phage synchronized with *C. difficile* administration was sufficient as prophylaxis against the infection; phage treatment post-infection saved 11 of 12 mice, whereas control animals administered with C. difficile and clindamycin died within 96 h [148].

Phage combinations also lowered *C. difficile* growth significantly in in vitro and limited proliferation in vivo using a hamster model [149]. Intraperitoneal administration of a single phage strain was effective to rescue 100% of mice in bacteremia models using vancomycin-resistant *E. faecium* [150], extended-spectrum β-lactamase producing *E. coli* [151], and imipenem-resistant *P. aeruginosa* [152].

Phage cocktails can be utilized in treating antibiotic-resistant *P. aeruginosa* infections of the skin, lungs, and gastrointestinal tract in animal models [153,154]. Reports also suggest that phages have the potential to restore sensitivity in antibiotic-resistant bacteria like the case of multidrug-resistant *P. aeruginosa*. Phages are combatting antibiotic-resistant bacteria by limiting their capacity to evolve resistance [155,156].

Unlike antibiotics, phages may evolve novel counter-defense mechanisms to counter bacterial resistance at a rate that can never be replicated by researchers developing antibiotics [157,158,159,160].

*A. baumannii* is a nosocomial pathogen that is rapidly evolving resistance against antibiotics. Two novel bacteriophages, named PBAB08 and PBAB25, were used against the MDR *A*. *baumannii* in a mouse model. Mice treated with a phage cocktail showed a 2.3-fold more survival rate along with a 1/100 reduction of the total number of A. baumannii in the lungs [161]. A person infected with MDR *A*. *baumannii* was treated with bacteriophages, and the patient was reported to be more alert than before, his craniotomy site and skin flap healed very well. He had lost all the symptoms of the infection and got healthy [162]. These findings suggested that the newly isolated phages could be used as effective therapeutic candidates against (MDR) *A. baumannii*.

Phage lysins are solely capable of lysing bacterial cells, and they have been identified as potential antimicrobial agents. These proteins are efficient, potent, and inactive against eukaryotic cells. Mice have been successfully saved from bacteremia through lysins caused by MDR *A. baumannii* [163], *Streptococcus* [164], and MRSA [165].

Using phage lysins and antibiotics in amalgamation may prove more effective at eradicating infections than by using antibiotics solely, as displayed in vitro and in vivo in a colon model using *C. difficile* [166]. All lysins do not show equal therapeutic potential, however as highlighted in an article [167], a highly potent lysin, PlySs2, was identified, which proved very effective against several pathogenic *Streptococcus* and *Staphylococcus* species, such as MRSA, and remained fully operational even after 10 freeze-&-thaw cycles. A recent study about the isolation and application of phage proteins has shown that lysins can cross the epithelial cell membrane to eradicate intracellular infections of *S. pyogenes* [168]. Phage lysins also interrupt vegetative cells, as displayed with *B. anthracis* lysin PlyG, which has the potential of attacking endospores of *Bacillus*, a major advantage over antibiotics. Table 4 enlists and summarizes different phages and phage cocktails used against resistant bacteria.

## 8. Immune Stimulation via Bacterial Extracts

Bacteria and bacterial extracts have been used for immunotherapy for several years. Recently, it has been discovered that these nonspecific immune activators trigger specific receptors of immune cells (and certain molecular signaling pathways); opened a new era of targeted immunotherapy. It can be achieved by using chemically synthesized molecules mimicking specific pathogen molecules. Bacterial extracts contain specifically and nonspecifically stimulating agents that activate innate and the adaptive immune system [171]. For the treatment of complicated as well as (other) infections that are caused by resistant strains, bacterial extracts can be used as adjuvants [172]. Bacterial extracts can activate macrophages and monocytes due to the presence of bacterial wall structures, like lipopolysaccharide or proteoglycans, which interact with Toll-like receptors (TLR) that are expressed over the surface of monocytes. Due to this interaction, monocytes are activated; they will differentiate into immature dendritic cells and then mature into dendritic cells, which are considered as suitable antigen-presenting cells, and the activation of this mechanism would cause a stimulation of the immune response. The antigen presentation over mature dendritic cells, in turn, stimulates T helper and B lymphocytes, following the maturation into plasma cells along with antibody production. These antibodies then cause bacterial opsonization, followed by destruction via macrophages [173].

Lipopolysaccharide (LPS) is a well-known virulence factor of bacteria that stimulates an innate immune response in hosts. In one study, LPS was extracted from the highly resistant isolates of *Proteus mirabilis* and incorporated into a liposomal delivery system. It was injected in rats via the intraperitoneal route, and its efficiency in stimulating immune responses was weighed by determining the Toll-like receptors and CD14 levels. The results showed that liposomes having incorporated LPS could release moderate levels of Toll-like receptors-4 (TLR4) that, in turn, enabled the immune system to clear pathogens [174].

## 9. Vaccination

Vaccination had been so fruitful in the world of multidrug resistance owing to its mechanism of action that diminishes the burden of the disease, thereby reducing the use of antibiotics and thus culminating the basis of MDR evolution. Since antibiotics are not being utilized (in this case), the resistance cannot develop, and selection cannot occur; neither of the pathogen at hand nor the “bystander” species present [175,176]. The vaccination process can considerably influence the MDR better if herd immunity is conferred [175,177], that is, protection of unvaccinated population by the vaccinated population such that they act as buffers, not being affected by the disease themselves and thereby reducing the chance of transmission to others. Moreover, for vaccines against bacteria like *S. pneumoniae*, *S. aureus*, and members of the family *Enterobacteriaceae*, which inhabit the nasopharynx, skin, gastrointestinal tract; there is the hypothetical likelihood that plummeting the density of bacterial populations by vaccination diminishes the prospects for genetic reassortment and recombination of resistant genes [175,178,179]. Vaccines that are generated specifically against virulence factors can be very powerful because these days, there are numerous genomic sequences available for almost all species. The state-of-the-art technologies and methods, such as reverse vaccinology, which can filter out the best models, are suited for enhanced immune responses. Some vaccines that are currently under production directing the aim towards virulence factors: (i) a tetra-subunit vaccine comprising of two capsular polysaccharides and two virulence-associated proteins (ClfA and MntC) against *S. aureus*, which is presently in phase 2b trials [175,180], (ii) three vaccines against *C. difficile* constructed on toxins A and B which are in phase 2 and 3 trials [181], (iii) a vaccine against *P. aeruginosa* founded on conserved outer membrane protein F/I fusion which is in phase 2/3 trials in an intensive care unit (ICU) patients [175,182], and (iv) a vaccine for *Candida* targeting T cell target protein, Als3 [175,183,184] is in the phase 2 trials. In recent times, it has been anticipated that directing vaccines against already resistant strains or even against resistance determinants themselves may be an actual way to thwart the selection pressure for antimicrobial resistance [23,175,185]. Antiresistance vaccines ought to be more operational against the drug-resistant strains in comparison to drug-susceptible ones by explicitly targeting resistant alleles of a conserved protein (for instance, a neuraminidase binding protein in the influenza virus) or by targeting proteins exclusively present in resistant clusters (such as ribosomal methylases deliberating macrolide resistance). Two vaccines are being developed under the shadow of this theory; that is, the resistance causing elements can be the foundation of vaccines providing a strong immune response. Protection against MRSA [175,182,186,187] can be sought through a vaccine that aims for the resistance-conferring extra penicillin-binding protein (PBP2a), while in *Neisseria meningitidis*, the target would be one of the essential penicillin-binding proteins [175,188]. The use of a variety of vaccines that may provide immunity against infectious agents is summarized in Table 5.

## 10. Combination Drug Therapy

Combination therapy is when a set of drugs are used to treat infections rather than a single drug (monotherapy). Infections caused by causative agents (*M. tuberculosis*, human immunodeficiency virus, *Plasmodium* parasite), which are predisposed to develop resistance, are being treated with this method [147]. The use of combination drug therapy acts in multiple dimensions. Recently, for the treatment of gonorrhea, which recommends ceftriaxone or cefixime plus azithromycin [191].

### 10.1. Combination Drug Combination Acting on Diverse Targets in Different Pathways

A classic example is a treatment modality used against *M. tuberculosis* infections currently prevalent in many developing nations like India. Four first-line drugs are used in this regimen: rifampicin (R), isoniazid (H), ethambutol (E), and pyrazinamide (Z); their targets are rifampicin (RNA polymerase inhibitor), isoniazid (enoyl reductase subunit of fatty acid synthase), ethambutol (an inhibitor of arabinosyl transferases involved in cell wall biosynthesis) and pyrazinamide (mechanism of action poorly understood) [147,192,193]. This method is highly effective since a bacterium may develop resistance by changing one of its targets; the combination drug strategy will still be effective against at least the other two pathways minimizing the chances of bacterial propagation.

### 10.2. Drug Combinations Acting on Diverse Targets in the Same Pathways

β-lactamase enzyme produced by Gram-positive bacteria opens up the β-lactam ring making it non-functional. Thus, this approach involves the use of a β-lactam antibiotic (amoxicillin) and β-lactamase enzyme inhibitor (clavulanic acid) [147,187]. Clavulanic acid degrades the enzyme, allowing the drug to destroy these microorganisms.

### 10.3. Drug Combination Acting on a Single Target, but in Multiple Dimensions

Streptogramins are made up of two active molecules that bind to the adjacent sites in the 50S ribosomal subunit near the peptidyl transferase center [194,195]. When both of these molecules are used simultaneously, they show 10–100-fold more potency as compared to using a single molecule alone [196]. Table 6 defines different combinations of drug therapy.

## 11. Novel Antibodies against MDR Bacteria

The conception of monoclonal antibody therapy against MDR superbugs stems from the fruitful usage of serum therapy against bacterial infections. The effectiveness of this technique was authenticated in the clinical trials and is in medical practice since the early 1900s entailing a particular antiserum serving as antimicrobial agents. Serum therapy was aborted with the advent of antibiotics, in some measure, owing to frequent toxicities and the failure to refine or generate antibodies for single determinants back then. Conversely, in the present day, technological and research progress in antibody engineering makes it conceivable to produce distinct, consistent, and completely human (humanized) mAbs with a particular antigen specificity.

A1102 is a humanized mouse gal-III mAb whose biologic activity in vitro and in vivo was demonstrated in experimental models of *K. pneumoniae* ST258 infections. It was revealed that upon passive immunization with A1102 before infection with ST258 whole bacteria or ST258-derived LPS increased the survival rate of endotoxin-sensitized mice and also protected rabbits from a lethal infection with ST258. It was shown by in vitro studies that the biological action of A1102 comprising complement and Fc independent LPS neutralization necessitated divalent binding and augmentation of human serum bactericidal eradication and complement-dependent macrophage uptake of ST258 [197].

**Figure 8:**
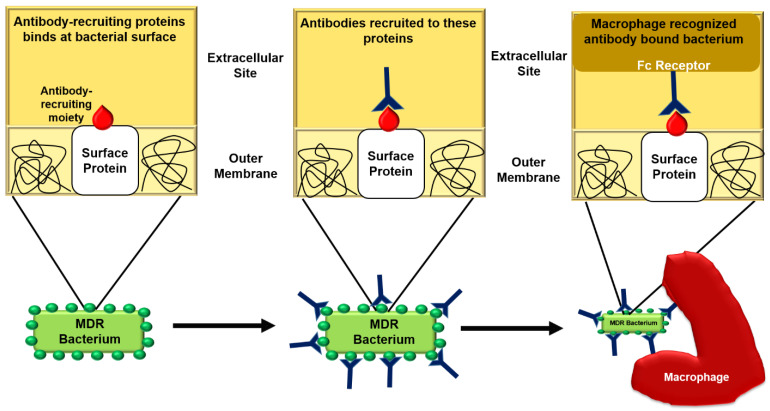
Mode of action of novel antibodies how they bind to multidrug-resistant (MDR) bacteria and present to macrophages and destroy them.

It had been demonstrated that O25b-specific MAb ASN-4 retained its bactericidal activity against an *MCR-1*-positive colistin-resistant ST131-H30 strain by three mechanisms of action that are opsonophagocytosis, endotoxin neutralization, and complement-mediated killing. Subsequently, LPS O-antigen-targeting antibodies are thought to be an alternative way of combating MDR infections, including the emerging *MCR-1*-positive isolates [198]. The general mode of action of novel antibodies against bacteria is depicted in Figure 8.

Even though antibody-based approaches of prophylaxis may have substantial potential for the inhibition of bacterial infections, their conspicuous operating principle may correspondingly counterpart antibiotics. Combination drug therapy is broadly considered to diminish the occurrence of resistance, but antibacterial antibodies have likewise been established to deliver assistance when used alongside antibiotics comparative to management with antibiotics single-handedly. For instance, mice doctored with a comparable human dosage of tobramycin did not endure infection with a tobramycin-resistant *P. aeruginosa* experimental isolate; however, therapy with tobramycin and a sub-protective MEDI3902 (bispecific antibody targeting the *P. aeruginosa* type III secretion (T3S) protein PcrV and Psl exopolysaccharide) dosage ensured in mice survival and improved bacterial clearance. Comparable advantages have been described employing using mAbs adjunctively with antibiotics against bacterial infections [199]. Table 7 defines different antibodies against bacteria.

## 12. Carbon Monoxide-Releasing Molecules (CORMs)

Studies have been conducted on animal models regarding the usage of hem oxygenase activity intracellular product called carbon monoxide (CO), and its administration as a therapeutic agent showed beneficial effects in treating animal models suffering from inflammatory disorders and cardiovascular diseases as well as in the organ transplantation [200].

However, the same administration of CO in humans can be hazardous as the levels of systemic carboxyhemoglobin can be raised high enough to cause death. Prodrugs such as carbon monoxide-releasing molecules (CORMs) transfer CO into biological systems. They are considered a safer alternative since they do not affect the transport of oxygen by hemoglobin.

Among all the CORMs reported up to now, metal carbonyl complexes are the most suitable and popular class of compounds. Some other CORMs include oxalates, tertiary aldehydes, silacarboxylates, and boron carboxylates [201]. When the carbonyl groups are attached to the transition metals such as molybdenum, iron or ruthenium, the CORMs exhibit a unique ability to transfer CO in the cells and enhance the signal transduction mechanisms mediated by CO. Thus, CORMs, as donors of CO, presented an extensive range of biological activities. Moreover, some specific transition metal carbonyls have repeatedly presented cytoprotective properties as well as some curative activities in a wide range of cellular and in vivo models of diseased animals [202,203].

## 13. Probiotics

Probiotics are known to be live microorganisms, primarily belonging to the genus *Lactobacillus* and *Bifidobacterium*, which are well-known to have a very advantageous influence on the host organism they inhabit. Concerning antibiotics and MDR, probiotics are seen to participate in diminishing the threat of various infectious diseases, including gastroenteritis and respiratory tract infections. Simultaneous utilization of probiotics with antibiotics has been proven to lessen the occurrence, time period, and/or ruthlessness of antibiotic-linked gastroenteritis. This, in turn, has better effects on the adherence properties of the antibiotic, thereby enhancing the efficacy of these antibiotics. *Lactobacillus* strains are also known to be successful in the defense of the host against urinary tract infections. *L. rhamnosus* GR1 has an amazing capability to bind with epithelial cells, particularly in the vaginal tract and is not susceptible to spermicidal agents; therefore, the bacterium can avert binding and development of urinary microorganisms [204]. *P. aeruginosa* has intrinsic resistance to the majority of accessible antibiotics, including aminoglycosides, anti-pseudomonal penicillins, newer cephalosporins, and imipenem. The favorable effect of probiotics may be linked to their aptitude to inhibit the development of drug-resistant bacteria seemingly by the secretion of antibacterial chemicals including lactic acid, hydrogen peroxide, and more diminishing their ability to colonize the body, thereby reducing the use of antibiotics and therefore, the development of MDR as a whole [20,21].

In one current study, indigenous probiotic *Lactobacilli* and standard *Lactobacillus* strains were evaluated for their inhibitory activity against MDR *K. pneumoniae*. Probiotic lactobacilli strains; *L. plantarum* LMEM7, *L. rhamnosus* LMEM9, *L. acidophilus* LMEM8, and *L. animalis* LMEM6 isolated from curd samples and *L. fermentum* MTCC 9748 standard strains were used against *K. pneumoniae*. Results obtained showed that indigenous *Lactobacilli* could be used against MDR *K. pneumoniae* in place of antibiotic therapy, and more probiotic strains should be identified against different pathogens [205]. In a similar study, the antimicrobial effect of *Propionibacterium freudenreichii* derived from dairy had been identified against multidrug-resistant *Salmonella heidelberg* (SH) in turkey poults. Two strains were used; namely, *P. freudenreichii freudenreichii* B3523 (PF) and *P. freudenreichii shermanii* B4327 (PS). The analysis revealed that *P. freudenreichii* could be used as an alternative to antibiotics for preventing SH infections in poults [206]. Table 8 indicates probiotics use to combat bacteria.

## 14. Quorum Sensing

Bacteria interact with each other through small chemical molecules (in a coordinated manner) known as quorum sensing. The buildup of quorum-sensing signals (QSS) in the growth medium reflects cell density, and as soon as a viable concentration is achieved, the QSS trigger transcription factors that in turn upregulate the signal synthase and several other genes. It is accepted that QSS govern virulence factor manifestation, which is our main interest coupled with bioluminescence, making biofilm, admission into stationary phase, sexual conjugation, sporulation, and transformation capability [22].

**Figure 9:**
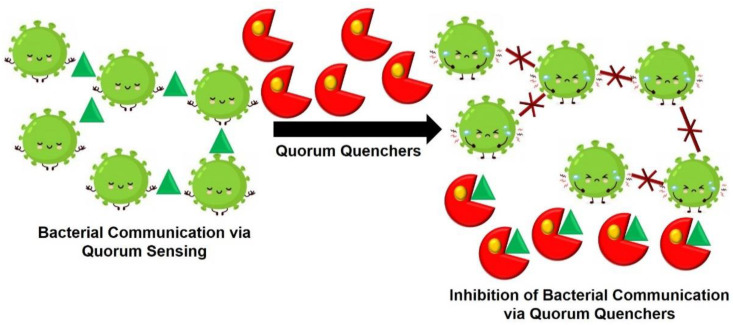
Quorum sensing can be blocked by producing and apply chemical analogs, which render the bacteria unable to communicate and hence pathogenicity-related expression is affected.

A huge number of hospital-acquired ailments are associated with infections instigated by biofilm molded on implanted devices. The consequence of these infections can be lengthier hospitalization, surgical operations, and even demise. A peptide molecule is known to hinder biofilm formation and ailments caused by *S. aureus*, which somehow blocks the quorum sensing (coordination) between bacteria. The heptapeptide, which was initially isolated from post exponential supernatants of *S. xylosus*, is now made in its amide form as a synthetic 7-aa molecule (YSPWTNF-NH2) termed “RNAIII-inhibiting peptide” (RIP). RIP inhibits cell adhesion and biofilm formation; the activity of the gene locus *agr*, thus preventing the production of a regulatory RNA molecule, RNAIII, that controls the production of toxins and affects the pathogenesis of *S. aureus* [135,136]. The mechanism through which RIP inhibits quorum-sensing mechanisms includes inhibition of the phosphorylation of a protein called “target of RNAIII activating protein”. The antibiotic concentration essential to destroy bacteria in the biofilm is 100–1000 times greater than that required to kill the same species outside a biofilm. The greater the use of antibiotics, the more the chances of resistance development against them. Thereby, by reducing the use of antibiotics achieved via quorum sensing inhibition, we can effectively prevent MDR in bacteria [135,207]. Figure 9 represents the mode of action how a quorum quencher can interfere with bacterial communication leading to biofilm inhibition.

## 15. Vaccines vs. Drugs: Who Is Going to Win?

Vaccines are majorly used as prophylactics as opposed to drugs. Vaccines confer immunity to individuals from the pathogens at hand, thereby preventing it from reaching massive numbers in the body, which in turn diminishes the buildup of genetic diversity or recombination frequency as well as culminates any chances for further communication to other hosts. For instance, a tuberculosis vaccine has been reported to reduce a bacterial population peak size up to five times the actual number attained in rodent subjects [24,208]. Furthermore, vaccines are designed as such to encompass more than one pathogenic antigen as well as multiple epitopes for the immune system to recognize and develop memory against [24,209]. Epitopes are recognized and processed by apparatuses of the immune system comparable to the method of biochemical molecules interacting with a drug. This shows that the immune response is actually like combinational drug therapy. With significant additional component effectors, however, it is revealed that roughly 100 distinctive tetanus-toxoid-specific antibodies can be witnessed in hale and hearty humans after getting a tetanus-toxoid booster vaccine, with distinct antibodies from one individual to another [24,209,210,211].

## 16. How Important Is the Accurate and Rapid Detection of the MDR Bacteria?

As already discussed in detail that antimicrobial resistance is a worldwide issue that leads to morbidity and mortality. This situation can be resolved by developing rapid diagnostics tools for quick profiling of pathogens, and their resistance [212]; one-way to fight this issue is by prescribing accurate antibiotics. This can be done by proper and rapid differentiation of bacterial and viral infections, and it is difficult to achieve because pathogen isolation, identification, and antibiotic resistance detection take time. This can be resolved by implementing point-of-care (POC) diagnostics. There are three classes of POC diagnostics; one that differentiates between bacterial and viral infections; second, in which detection and report of a specific pathogen are performed, and the third one is, it should not only detect and report a specific pathogen but also exhibit the presence of antibiotic resistance [213].

Many bacterial detection techniques, like infrared light-based devices, polymerase chain reaction (PCR), enzyme-linked immunosorbent assay (ELISA), and chemical assays, are available but are slow and require sophisticated equipment. Culturing is considered the gold standard for the detection of bacteria, but it requires several days to retrieve the final results. Hence, there is a need for those techniques that are not only fast but also sensitive in detecting bacteria. In a new method called whispering gallery mode (WGM), optical microcavity-based sensors were used to detect the bacteria *Helicobacter hepaticus*. It was shown that this bacterium could easily be detected using WGM optical microcavity-based sensors [214]. WGM sensors can react to environmental perturbations. Their sensitivity, together with the diversity of structures, leads to the development of these devices for a wide range of analytes. These are being used for detecting clinically relevant biomolecules as well as single-protein interactions [215]. Thus, this technique is proven to be faster and potentially sensitive.

Optical (bio)sensors facilitate us by monitoring polarization, intensity, phase, speed, and frequency of light. Whispering gallery mode (WGM) microresonators are at the forefront for over the last two decades [216]. They provide a label-free optical method to detect bacteria rapidly with high sensitivity [217]. They are increasingly used as transducers for detecting specific biomolecules. They can detect biomolecules in a label-free manner without any chemical modifications of the analyte. Additionally, the biophysical properties of biomolecules do not get altered due to probing light. WGM sensors detect biomolecules by recognizing the target analyte through molecular receptors. Thus, WGM frequency shifts that are specific to the analyte molecule are produced. Biomolecular detection is achieved by analyzing the binding of analyte molecules to the receptors and converting the binding event into optical and electrical signals, which are then detected and measured as spectra over time. Receptor molecules that are being used in WGM biosensing are oligonucleotides, antibodies, and proteins [218]. Due to the small mode size of the microcavity, the light which enters the microcavity can cycle multiples of times under TIR (total internal reflection) action. In the meantime, this light extends in the surrounding medium in the form of evanescent waves, and the intensity of the evanescent wave falloffs exponentially. Mode change occurs as a result of any interference in the evanescent field, and this results in substantial variations in the resonant characteristics like mode separation, frequency shifting, and linewidth broadening. This causes the enhancement of interaction between light and matter, which results in realizing unprecedented sensitivity detection [219].

## 17. Conclusions

Bacteria are naturally programmed to survive, and it is why they had always been one step ahead of humans. Nevertheless, it is high time to gain the advantage over these menacing creatures by the advent of modern science. To gain an edge over these MDR microorganisms, we must consume our time and energy towards rapid diagnostic systems to equip us with the knowledge of what we are dealing with. Moreover, it is proved through literature that approaches that have nonprotein targets in bacteria are the most promising ones as the bacteria have so far not been able to cope up with it efficiently. To end this continuous war, we must also keep on isolating new drugs with novel modes of action. 

## Figures and Tables

**Table 1:** Use of different nanoparticles as a weapon against drug-resistant bacteria.

Agent	Target Bacteria/Diseases	Mode of Action/Description	Notes (Advantages (a), Limitations (l), Combination Strategy (c))	Ref.
Nanoparticles				
AuNP with cationic surface chemistry	Gram-positive and Gram-negative bacteria	Interaction with cell membrane → formation of aggregates → bacterial cell lysis; cause protuberance	(a) Unique electronic, sensing, optical, and biochemical properties	[34,35,36,98]
CuONP	Variety pathogens, including MRSA, *E. coli*, *S. aureus*, *P. aeruginosa*, *N. meningitis*, *B. cereus*, *S. pyogenes*, *A. baumannii*	ROS → induce oxidative stress	(c) Antibacterial activity enhanced by conjugation with AgNPs (photocatalytic activity attributed to the production of ROS)	[1,38,39,40,99]
ZnONPs, colloidal ZnO suspension	MRSA, *S. agalactiae*, MRSE, MSSA, ESBL-producing *E. coli* and *K. pneumoniae*, *Vibrio cholera*, *Campylobacter jejuni*, *E. faecalis*, *S. epidermidis*, and other clinically relevant pathogens	Disorganization and damage of cell, cell membrane after internalization; damage of proteins, lipids, and DNA via oxidative stress	(l) Level of toxicity concentration dependent (c) Antibacterial activity enhanced by conjugation with AgNPs	[1,41,42,43,44,45,46]
Nitric-oxide-releasing NPs (NONPs)	Antibiotic-resistant and sensitive bacteria, i.e., *K. pneumoniae*, *E. faecalis*, *S. pyogenes*, *E. coli*, and *P. aeruginosa*	Formation of cell toxic reactive nitrogen and oxygen intermediates, NO-associated lipid damage, iron depletion, inhibition of DNA repair enzymes	(a) NO is unstable so spontaneously generate reactive intermediates	[47,48,49,50,51,52,53,54]
Gold nanorods	*P. aeruginosa*	Conjugated with primary antibodies	(a) Eradicate biofilms (c) 1,2-Distearoyl-sn-glycero-3-phosphorylethanolamine (DSPE)	[60,69]
	*S. aureus* and *Propionibacterium acnes*	Local hyperthermia by laser beam excited functionalized gold nanorods	(a) Enhanced reduction of viable bacterial count	[61]
AgNPs, colloidal AgNPs	Gram-positive and Gram-negative bacteria; drug-susceptible strains including *Streptococcus* spp., *E. coli*, and *P. aeruginosa;* MRSA, MRSE, (erythromycin-resistant) *S. pyogenes*, (ampicillin-resistant) *E. coli*, MDR *P. aeruginosa*	AgNPs anchor to cell wall leading to increased cell permeability by structural changes → uncontrolled transport through cell membrane; Membrane damage caused by AgNP produced free radicals; released Ag^+^ ions inactivate enzymes by interacting with thiol groups of enzymes	(a) Bactericidal against Gram-positive as well as Gram-negative bacteria (1) Dose-dependent	[55,74,75,76]
Biosynthetically produces AgNPs using fungus, yeast, bacteria, and plant extracts	*M. tuberculosis*, *P. aeruginosa*, *S. pneumoniae*, MRSA, *K. pneumoniae*, MRSE, *S. pyogenes*, *Bacillus* spp., *E. coli* and *S. typhi*	Inhibit cell wall synthesis, protein synthesis, which is mediated by the 30 s ribosomal subunit, and nucleic acid synthesis	(a) Strong antibacterial efficacy against various MDR pathogens	[81,82,83]
Aluminum oxide NPs	*E. coli*, *Pseudomonas fluorescence*, *Staphylococcus aureus*, *Streptococcus mutans*, *Proteus vulgaris*)	Disruption of bacterial cell wall by producing ROS, Serve as radical scavengers leading to distortion in bacterial cells	(a) Thermodynamically stable over a wide range of temperatures	[88,89,90]
Ethionamide (ETH)-conjugated SiNPs (silicon)	Multidrug-resistant *M. tuberculosis*	Enhance solubility and permeability of ETH at different pH-values	(a) Reduction of dosing frequency of ETH for the treatment of multidrug-resistant *M. tuberculosis* (c) thermally carbonized-porous silicon (TCPSi) loaded with ethionamide (ETH)	[94]
Gallium (III) nano-formulations	Drug-resistant *M. tuberculosis*	Targeted drug delivery, Promotion of maturation of phagosome → increased macrophage-mediated killing, Interruption of iron-mediated enzymatic reactions	(a) Active against resistant bacteria like *M. tuberculosis*, HIV	[96]
Nano-photothermal therapy				
AuNPs	Gram-positive bacteria, Gram-negative bacteria	Electromagnetic radiation absorbed by the NPs converted into heat → transferred via thermal conduction to bacteria in close proximities	(c) Conjugated with vancomycin or amoxicillin	[62,63,67]
Au nanorods	Pathogenic *E. coli*	Generate heat that lyses bacteria	(c) Heat generated by using continuous-wave laser irradiation or near-infrared laser	[29]
Au nanorods	*P. aeruginosa*	Nanorod attach to the bacterial cell surface allows the cell to expose to near-infrared radiation	(c) Conjugated with primary antibodies	[1,69]
Multifunctional popcorn-shaped magnetic iron core-shell gold nanoparticles	*Salmonella* DT104	Selective and irreparable cellular-damage	(c) Conjugated with *Salmonella* DT104 specific antibody	[70]
Polysiloxane polymers containing embedded methylene blue and AuNPs	MRSA and *E. coli*	Light-induced production of singlet oxygen and other reactive oxygen species by the methylene blue and gold nanoparticles enhanced activity of methylene blue	(a) Significant reduction of viable cell count (1) Require exposure to light and polymer formation	[1,71,72]

**Table 2:** Host defense peptides (HDP) against antibiotic-resistant bacteria.

Category	Agent	Target Bacteria/Diseases	Mode of Action/Description	Notes (Advantages (a), Limitations (l), Combination Strategy (c))	Status	Ref
HDP	Brevinin1 HYba1 and brevinin1 HYba2	Several Gram-positive and Gram-negative bacteria	Hemolytic, cytotoxic and antibacterial activities	(a) Very low hemolysis on human erythrocytes	Hep 3B cancer cell line	[101]
HDP (Defensin)	68 fungal defensin-like peptides (fDLPs)	A variety of bacterial, fungal and viral pathogens	(i) Cationic amphipathic peptides having approximately 30 amino acid residues (ii) From five genera named *Apophysomyces*, *Trichosporon*, *Scedosporium*, *Beauveria*, and *Lichtheimia* had been reported	(a) Higher antibacterial potential with lower cellular toxicities	In vitro and in vivo	[105]
	Scedosporisin (synthetic defensin)	Gram-positive bacteria, vancomycin-resistant *Enterococci*, MRSA	Scedosporisin-2 killed bacteria more rapidly as compared to the antibiotic vancomycin	(a) Low cytotoxicity and hemolysis on human	In vitro	[105]
HDP (Cathelicidin)	Bactenecin, indolicidin, protegrins,…	*S. pyogenes* and MRSA, VISA, *Listeria*	Produced by the immune systems of bovine and porcine	(a) Broad bacterial lytic properties, stability and higher efficacy	In vitro and in vivo	[106]
	Human cathelicidin LL-37	Antibiofilm activity against *S. aureus* and *E. coli*	Human immune system is known to produce only one type of cathelicidin precursor protein, hCAP18 → processed proteolytically to produce mature LL-37	(1) Exact mechanisms of interaction between LL-37 and immune cells have not been yet clarified	In vitro and in vivo	[107]
	CATHPb1–6 (six novel cathelicidins identified from *Python bivittatu*)	*S. aureus* (MRSA/VRSA)	Involved in modulating macrophages/monocytes; trafficking neutrophils to the site of infection and also enhance their bactericidal functions; increases levels of chemokines and reduces release of proinflammatory cytokines	(a) Provides protection via different administration routes	In vitro and in vivo (mice)	[108]

**Table 3:** A list of different antimicrobial peptides (AMP) (antibiofilm peptides (ABP)) molecules that can be used against multidrug-resistant (pathogenic) bacteria.

Category	Agent	Target Bacteria/Diseases	Mode of Action/Description	Notes (Advantages (a), Limitations (l), Combination Strategy (c))	Status	Ref.
ABP	Nisin	Variety of Gram-positive	Cationic peptide	(1) Requires proper optimization of pH	In vitro	[114]
ABP + HDP	Combination of peptides and defensin proteins	Variety of Gram-positive and Gram-negative bacteria; effective against fungi too	Proteinaceous entities can either be cationic or amphipathic	(a) Not require any specific protein binding sites	In vitro	[113]
ABP	Antimicrobial peptides KT2 and RT2	Antibiofilm activity against MDR enterohemorrhagic *E. coli* O157:H7	Tryptophan-rich cationic peptides permeabilize bacterial cell membranes → lead to death of cells by causing large damage or small obstructions that disturb transmembrane potential	(a) Not only prevent biofilm formation but also can eliminate mature biofilms (l) Interactions with membrane and each other (c) Combination with other antimicrobial compounds to enhance activity → lower concentration of antimicrobial compounds	In vivo	[100,120]
	Agelaia-MPI and Polybia-MPII	MDR *Acinetobacter baumannii*, several Gram-positive and Gram-negative bacteria, Mycobacteria as well as fungi	Isolated from wasps; bactericidal activity along with antibiofilm activity	(l) Production costs (l) Peptidases and proteases lead to low stability of peptides in human serum → (c) Increased stability in combination with other molecules (e.g., polyethylene glycol)	In vitro	[129]
	Ocellatin-PT2–PT6	Opportunistic pathogen *Pseudomonas aeruginosa*	Ocellatin-PT3 inhibits proliferation of established biofilms by directly killing bacterial cells	(a) Novel antimicrobial agent(l) Works better in combination BS antibiotics	in vitro	[130]
QS + ABP	“RNAIII-inhibiting peptide” (RIP)	Biofilm formation and ailments caused by *S. aureus*	Inhibition of phosphorylation of “target of RNAIII activating protein” → quorum sensing inhibition, prevention of MDR in bacteria	(a) Inhibits cell adhesion and biofilm formation	In vitro and in vivo (cellulitis)	[135,136]

**Table 4:** Bacteriophages as a therapeutic option against bacteria.

Agent	Target Bacteria/Diseases	Mode of Action/Description	Notes (Advantages (a), Limitations (l), Combination Strategy (c))	Status	Ref
Bacteriophages					
ϕMR11, KP DP1, SA DP1, PA DP4, EC DP3	*E. faecalis*, *S. aureus*, *Klebsiella*, *A. baumannii*, *P. aeruginosa*, and *Escherichia coli*	Phages bind to specific receptors on bacterial cell surface → infects bacterial cells → production of endolysins that damage bacterial cell wall by hydrolyzing four main bonds of peptidoglycan, Rupture of outer membrane via complexes (spanins) (Gram-negative bacteria)	(a) Applied externally and internally; high affinity for specific bacterium (normal flora not attacked) only one administration (replicative nature); can survive in the gastric environment; minimal side effects → ecologically safe; frequency of bacterial resistance to phages significantly lower compared with resistance to antibiotics (l) Not very effective in mixed infections (narrow host range)	In vivo (mice)	[19,169]
Lytic phage strain (KPP10)	*P. aeruginosa*	Decreased numbers of viable P. *aeruginosa* cells in blood, liver, and spleen as well as levels of inflammatory cytokines in blood and liver	(c) Oral administration	Animal models	[153]
CD140	*Clostridium difficile-induced* ileocolitis	Phage administration prophylaxis against infection	(1) Specific against *Clostridium difficile*	hamster	[148]
ØCDHM1–ØCDHM6, ØCDHS1, ENB6 and C33, Ø9882, ØA392, and KPP10	*Clostridium difficile*, vancomycin-resistant *E. faecium*, extended-spectrum β-lactamase producing *E. coli*, imipenem-resistant and MDR *P. aeruginosa*	Treatment of gut-derived sepsis	(a) Specifically act against bacterial pathogens (a) Do not affect the natural bioflora (a) Safer to use in humans (1) They will be effective only if their favorable conditions exist	Hamsters and mice	[150,154]
OMKO1	MDR *P. aeruginosa*	Outer membrane porin M (OprM) of the multidrug efflux systems MexAB and MexXY as a receptor-binding site	(a) Specifically act against MDR *P. aeruginosa* (a) Alters efflux pump mechanism to make the bacterium more susceptible to drugs	In vitro	[154]
PBAB08 and PBAB25	*Acinetobacter baumannii*	Reduction of bacterial load, increase in serum IgE with a slight increase of GM-CSF, IL2, IL10, and IL17A	(1) Inoculated in a cocktail and require properly set optimal conditions	mouse	[163]
Mixture of three phages	*Campylobacter jejuni* and *C. coli*	Reduce bacterial colonization	(1) Acquisition phage to resistance	Poultry	[143]
PlyF307 (phage lysin)	MDR *A. baumannii*	Lysing of bacterial cells	(a) Inactive against eukaryotic cells	Mouse	[163]
Cpl-1 (phage endolysin)	*Streptococcus pneumoniae*	Reduced pulmonary bacterial counts and prevented bacteremia, systemic hypotension, and lactate increase as well as reduction of penicillin-susceptible pneumococci	(1) Specific against pneumococci	Mouse	[164]
PGHs (phage endolysins)	MRSA	The peptidoglycan hydrolase enzyme targets the conserved regions and can destroy a wide range of mutant cell walls of bacteria	(a) Active against mutant and resistant strains (a) Also can clear static biofilms	In vitro and in Mouse	[165]
PlyCD (prophage lysin)	*C. difficile*	PlyCD specifically targets the pathogenic *C. difficile* while not affecting other commensal bacteria	(c) Phage lysins in combination with antibiotics more effective than antibiotics alone	Ex vivo mouse colon model	[166]
PlySs2 (phage endolysin)	*Streptococcus* and *Staphylococcus* species, such as MRSA	Lytic activity	(a) High therapeutic potential compared to other lysins	Mouse	[167]
PlyC (phage endolysin)	*S. pyogenes*	Lysins can cross the epithelial cell membrane to eradicate intracellular infections	(a) Ability to traverse epithelial membranes	model membranes	[168]
PlyG (phage endolysin)	Bacillus anthracis	Interrupt vegetative cells; major advantage over antibiotics (attacking endospores)	(a) Separate domains to recognize spores and vegetative cells	In vitro	[170]

**Table 5:** Different types of vaccines used to fight the bacterial multidrug resistance.

Agent	Target Bacteria/Diseases	Mode of Action/Description	Notes (Advantages (a), Limitations (l), Combination Strategy (c))	In Vitro, In Vivo, Clinical Phase, Animal Model	Ref
Vaccines					
Tetra-subunit vaccine	*S. aureus*	Comprising of two capsular polysaccharides and two virulence-associated proteins (ClfA and MntC)	(c) Diminish the burden of the disease, thereby reducing use of antibiotics	Phase 2b trial	[175]
Three different vaccines	*C. difficile*	Constructed on *C. difficile* toxins A and B	(1) More research is required for proper optimizations of toxin-based vaccines, including development and use of novel adjuvants	Phase 2 and 3 trials	[189]
OprF/I fusion protein vaccine	*P. aeruginosa*	Founded on conserved outer membrane protein F/I fusion	(a) Produce rapid immune response in healthy volunteers	Phase 2/3 trials	[175,190]
Vaccine NDV-3	*Candida*	Targeting T cell target protein, Als3	(a) Also protects from intravenous as well as skin and soft tissue infection with *Staphylococcus aureus*	Phase 2 trials	[184]
Antiresistance vaccines	MRSA	Cloned internal region from transpeptidase domain from penicillin-binding protein (PBP2a) as DNA vaccine	(a) More operational against drug-resistant strains by explicitly targeting resistant alleles of a conserved protein or by targeting proteins exclusively present in resistant clusters	Mouse	[186]
Antiresistance vaccines	*Neisseria meningitidis*	Vaccination with purified recombinant PBP2 + passive immunization with anti-PBP2 rabbit IgG antibody	(a) This vaccine candidate has a conserved region that is present in all strains of *N. meningitidis* and targeted by protective antibodies	Mouse	[175,188]

**Table 6:** Combination drug therapy to defeat the superbugs.

Combination Drug Therapy				
Agent	Target Bacteria/ Diseases	Mode of Action/Description	Notes (Advantages (a), Limitations (l), Combination Strategy (c))	Ref
Combination drug combination acting on diverse targets in different pathways				
Rifampicin (R), isoniazid (H), ethambutol (E), and pyrazinamide (Z)	*M. tuberculosis*	Rifampicin (RNA polymerase inhibitor), isoniazid (enoyl reductase subunit of fatty acid synthase), ethambutol (an inhibitor of arabinosyl transferases involved in cell wall biosynthesis) and pyrazinamide (mechanism of action poorly understood)	(a) Method highly effective since a bacterium may develop resistance by changing one of its targets, the combination drug strategy will still be effective against at least the other two pathways	[147,192,193]
Drug combinations acting on diverse targets in the same pathways				
Clavulanic acid	*Gram-positive bacteria*	Degrades the β-lactamase enzyme, allowing the drug to destroy these microorganisms	(c) Use of a β-lactam antibiotic (amoxicillin) and β-lactamase enzyme inhibitor (clavulanic acid)	[187]
Drug combination acting on a single target, but in multiple dimensions				
Streptogramins		Two active molecules that bind to the adjacent sites in the 50S ribosomal subunit near the peptidyl transferase center	(c) Both of these molecules are used simultaneously; they show 10–100-fold more potency as compared to using a single molecule alone	[194,195,196]

**Table 7:** Novel antibodies are used against bacteria with and/or without drug combination.

Agent	Target Bacteria/Diseases	Mode of Action/Description	Notes (Advantages (a), Limitations (l), Combination Strategy (c))	In Vitro, In Vivo, Clinical Phase, Animal Model	Ref
Antibodies					
A1102 (humanized mouse gal-III mAb)	*K. pneumoniae* ST258	Passive immunization with A1102 before infection with ST258 → infection prophylaxis	(1) Efficacy and exact role for protection in vivo not understood	In vitro and in vivo in experimental models (mice)	[197]
O25b-specific mAb ASN-4	*MCR-1*-positive colistin-resistant ST131-H30 strain	Oopsonophagocytosis, endotoxin neutralization, and complement-mediated killing	(a) Multiple mechanisms of action	In vitro and in vivo in experimental models (Murine models)	[198]
MEDI3902	*P. aeruginosa*	Bispecific antibody targeting the *P. aeruginosa* type III secretion (T3S) protein PcrV and Psl exopolysaccharide	(c) In combination with drug therapy (antibiotics) deliver assistance when used alongside antibiotics	In vivo (mice)	[199]

**Table 8:** Different types of probiotics used to combat bacteria.

Agent	Target Bacteria/Diseases	Mode of Action/Description	Notes (Advantages (a), Limitations (l), Combination Strategy (c))	Status	Ref.
Probiotics					
*Lactobacillus* and *Bifidobacterium*	*E. coli*, *Salmonella*, *Helicobacter pylori*, *Listeria monocytogenes* and rotavirus	Lessen occurrence, time period, and/or ruthlessness of antibiotic-linked gastroenteritis → enhancing efficacy of these antibiotics	(c) Simultaneous utilization of probiotics with antibiotics	In vivo	[204]
*Lactobacillus acidophilus* strain	*P. aeruginosa*	Inhibit development of drug-resistant bacteria by secretion of antibacterial chemicals including lactic acid, hydrogen peroxide, diminishing MDRs ability to colonize the body → reducing use of antibiotics	(a) Reduced use of antibiotics and development of MDR by providing protection against intrinsic resistance strains	In vitro	[20,21]
*Lactobacilli*	MDR *K. pneumoniae*	Used in place of antibiotic therapy	(1) Require identification of more strains	In vitro	[205]
*Propionibacterium freudenreichii freudenreichii* B3523 (PF) and *P. freudenreichii shermanii* B4327 (PS))	MDR *Salmonella heidelberg* (SH)	Used as an alternative to antibiotics for preventing SH infections	(a) Non-host gastrointestinal tract-derived probiotic	Turkey poults	[206]

## Data Availability

Not Applicable.
